# The exchange bias behavior of BiFeO_3_ nanoparticles with natural core-shell structure

**DOI:** 10.1038/s41598-018-19676-5

**Published:** 2018-02-02

**Authors:** Fengzhen Huang, Xingyu Xu, Xiaomei Lu, Min Zhou, Hai Sang, Jinsong Zhu

**Affiliations:** 1 National Laboratory of Solid State Microstructures and Physics School, Nanjing University, Nanjing, 210093 P. R. China; 2 Collaborative Innovation Center of Advanced Microstructures, Nanjing, 210093 P. R. China

## Abstract

The surface and interface effects of small antiferromagnetic nanostructures are important on the modulation of their magnetic properties. In this report, temperature and particle size dependent magnetic exchange bias effect was investigated in BiFeO_3_ (BFO) nanoparticles that possess natural core-shell structure. Nonmonotonic variation of exchange bias field, interesting surface spin-glass state and improved exchange bias training effect are only obtained in 18 nm BFO particles. Based on comparative experiments on particles with different sizes, we found that the surface spins and the interaction among them show great effect on the interfacial exchange coupling of the core-shell structure, and thus are responsible for the peculiar exchange bias behavior in small BFO nanoparticles. Our work provides the effect of surface spin state on the magnetic characteristics of nanomaterials and will favor their applications on spintronic devices.

## Introduction

BiFeO_3_ (BFO) has received considerable attention as one of the most important single-phase multiferroics because of its high ferroelectric Curie temperature (*T*_*c*_) of about 1103 K and antiferromagnetic Néel temperature (*T*_*N*_) of about 643 K^1–6^. The magnetic structure of BFO is of G-type antiferromagnetic order with Fe magnetic moments aligned ferromagnetically within pseudocubic (111) planes and antiferromagnetically between adjacent (111) planes. The combined action of exchange and spin-orbit interactions produces spin canting away from perfect antiferromagnetic ordering, resulting in a spiral spin arrangement with a wavelength of about 62 nm^7–10^. While with the decrease of particle size, surface effect usually leads to a breaking of the sublattice pairing in antiferromagnet and results in a net surface magnetic moment. Therefore, BFO nanoparticles can be modeled by a superposition of an antiferromagnetic (AFM) core and a ferromagnetic (FM) surface^8,10–12^. The exchange coupling at the AFM/FM interface of a core-shell structure often leads to a phenomenon called “exchange bias”, which draws significant interests in recent years due to its important potential technological applications in various magnetic devices^13–18^. Recently, exchange bias effect was observed in BFO and its doped particles, and the results obtained by different research groups reveal that both the magnitude of the exchange bias field (*H*_*E*_) and its temperature-dependent behavior show obvious sample dependence^12,19–22^. While the origin of such complex sample-dependent behavior is still unclear and needs further investigation. In this paper, the exchange bias effect of BFO nanoparticles with different sizes is comparatively investigated. Temperature-dependent nonmontonic variation of *H*_*E*_ along with improved training effect is observed only in 18 nm BFO particles. Such interesting behavior is considered to be closely to its enhanced surface anisotropy and the presence of surface spin-glass state.

## Results and Discussion

**Figure 1:**
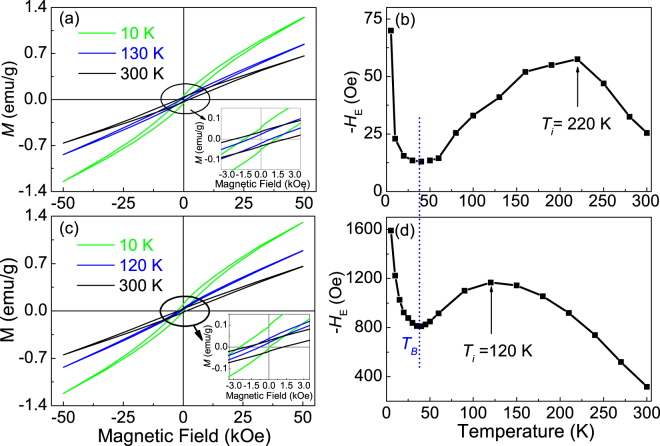
Magnetic measured results of 18 nm BFO particles at 50 kOe. Magnetic hysteresis (*M-H*) loops measured at various temperatures following (**a**) zero-field cooling (ZFC) and (**c**) 65 kOe cooling field. Temperature dependent exchange bias field (−*H*_*E*_) under (**b**) ZFC and (**d**) 65 kOe cooling field. Insets in (**a**) and (**c**) show the local amplification of the corresponding *M-H* loops.

The prepared nanoparticles present single-phase perovskite structure, and the diameter of the smallest particles is about 18 ± 5 nm^10^. Figure 1 shows the magnetic measurement results of the 18 nm BFO particles. It is seen from Fig. 1(a), which is measured under zero-field cooling (ZFC) condition, the magnetic behavior of the sample, such as coercivity (*H*_*c*_), remanent magnetization (*M*_*r*_), etc. show obvious temperature dependence. Moreover, the magnetic hysteresis loops exhibit a small shift along the magnetic field axes, implying the presence of an exchange bias effect in 18 nm BFO particles even in ZFC condition. The exchange bias field is calculated based on H_*E*_ = (*H*_*c1*_ + *H*_*c2*_), where ${H}_{c1}$ and ${H}_{c2}$ are the coercive fields at the descending and the ascending branches of the magnetic hysteresis loop, respectively. In order to get a clearer image of *H*_*E*_, its variation as a function of temperature in ZFC condition is shown in Fig. 1(b). With increasing temperature from 5 K, −*H*_*E*_ (−*H*_*E*_ is used to represent the absolute value of *H*_*E*_ since it is negative in the whole measuring temperature range) decreases sharply at first and reaches its minimum at temperature of about 35 K, and then increases with increasing temperature from 35 to 220 K while decreases with further increasing temperature. The exchange bias effect can be enhanced by cooling magnetic field (*H*_*cooling*_). As shown in Fig. 1(c,d), more obvious exchange bias behavior with larger −*H*_*E*_ is observed when measuring after cooling the samples under magnetic field of about 65 kOe. Similar with that obtained in ZFC condition, nonmonotonic variation of −*H*_*E*_ is observed with increasing temperature, while the inflection temperature *T*_*i*_ decreases from 220 K to 120 K under FC measurement. It should be mentioned that, though the loops shown in Fig. 1 seem unsaturated at 50 kOe, the calculated values of −*H*_*E*_ and its variation behavior with temperature are similar with that obtained under 65 kOe (supplementary Fig. S1), implying the ferromagnetic part of BFO nanoparticles has probably reached saturation when the applied magnetic field is larger than 50 kOe. It is known that BFO nanoparticles not only include ferromagnetic shell but also aniferromagnetic core, and both parts contribute to the loop measurement, therefore, saturation magnetic loops cannot be observed by increasing measuring field. While as shown in supplementary Fig. S2, the loops deducted the linear contribution of antiferromagnetic part are saturation at 50 kOe.

**Figure 2:**
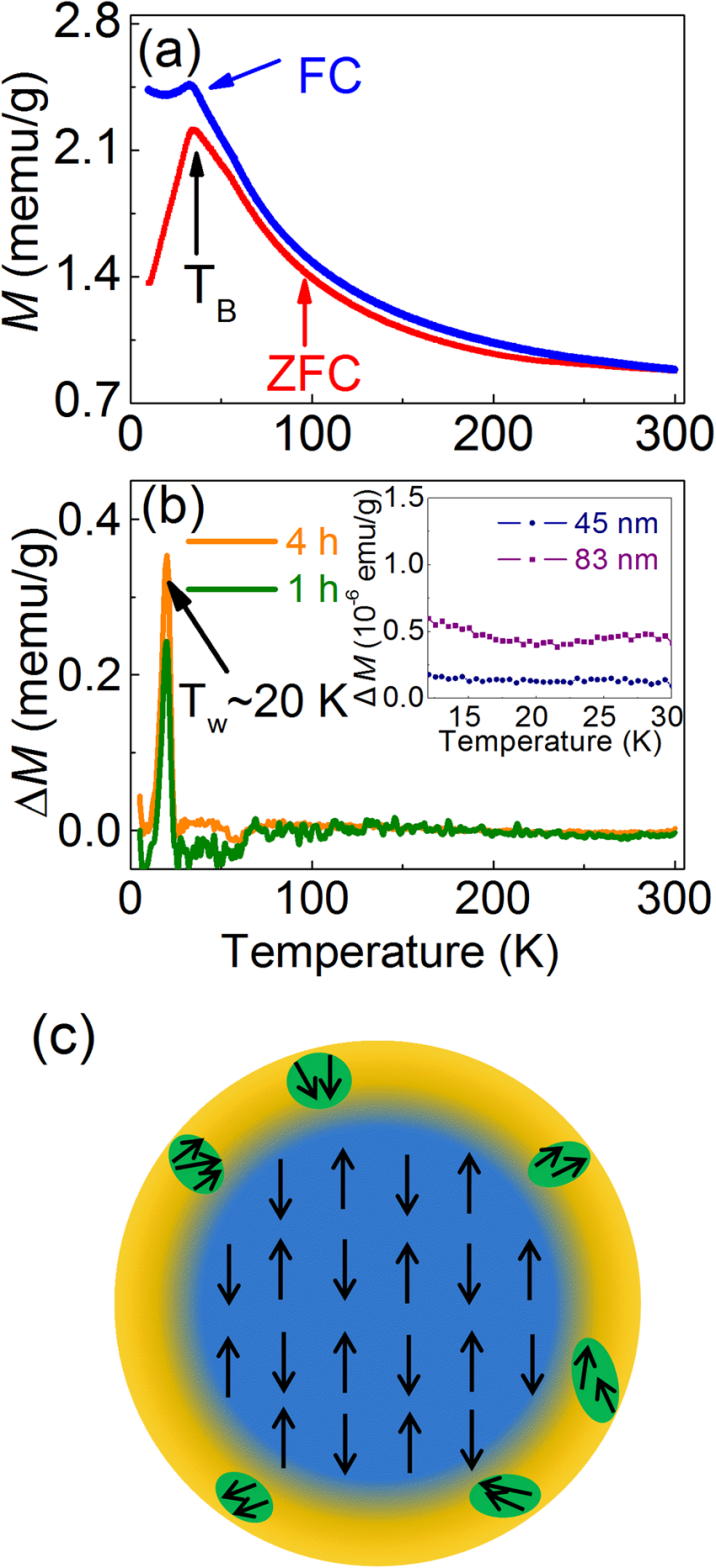
(**a**) ZFC and FC magnetization curves of 18 nm BFO particles measured under 50 Oe. (**b**) Magnetization difference $(\triangle M)$ between the reference and the stop-and-wait ZFC curves of 18 nm BFO particles. (**c**) Schematic diagram of the AFM/FM core-shell structure for BFO nanoparticles. The inset in (**b**) shows the $\triangle M({\rm{T}})$ of 45 and 83 nm particles, where T_*w*_ = 20 K, and the waiting time is 1 h.

**Figure 3:**
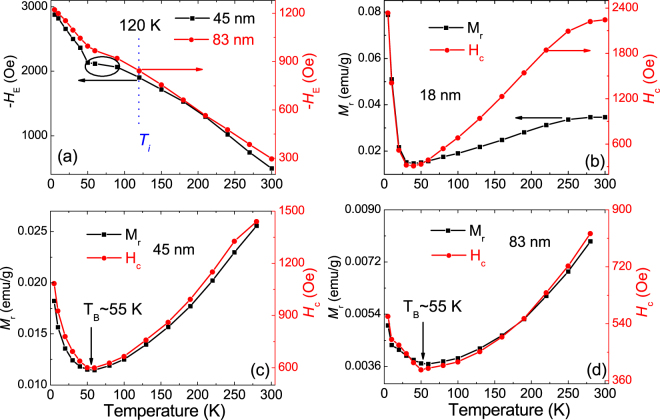
(**a**) Temperature dependent *−H*_*E*_ of 45 and 83 nm BFO particles that measured at 50 kOe after cooling under 65 kOe from 380 K. Temperature dependent *M*_*r*_ and *H*_*c*_ of BFO particles with size of about (**b**) 18 nm, (**c**) 45 nm and (**d**) 83 nm, which are calculated from the ZFC magnetic hysteresis loops. The *T*_*B*_ of 45 and 83 nm particles, which corresponds to the frozen temperature of their surface spins, is about 55 K, larger than the appearing temperature of spin-glass state (T_B_ ~ 35 K) of 18 nm BFO particles.

To trace the origin of the interesting variation of −*H*_*E*_, temperature dependent magnetization are measured for the 18 nm BFO particles. As shown in Fig. 2(a), a blocking temperature of about 35 K (*T*_*B*_), which corresponds to the minimum of −*H*_*E*_, is observed in the ZFC curve, and the FC curve flattens off below this temperature, implying the appearance of a possible spin-glass behavior. The magnetic memory effect below *T*_*B*_, a typical magnetic effect in spin glass state^23^, is explored by a stop-and-wait protocol to check the appearance of spin-glass state. First, the sample was cooled under ZFC condition down to a temperature *T*_*w*_, an arbitrary temperature below *T*_*B*_, and 20 and 25 K are selected in this report. As the temperature reached *T*_*w*_, the cooling process was paused for a period of time (1 h and 4 h in our experiments), and then resumed the process down to 5 K. Second, the magnetization (${M}_{{saw}}({\rm{T}})$) was measured under 200 Oe with increasing temperature from 5 K to 300 K. The magnetization difference $\triangle M({\rm{T}})$ at the temperature *T*_*w*_ between the stop-and-wait ZFC curve and the reference ZFC curve (in which ${M}_{{ref}}(T)$ was measured at the same measuring condition but without stopping at *T*_*w*_ under the cooling process) is calculated by $[{M}_{{ref}}(T)-{M}_{{saw}}({\rm{T}})]$ and shown in Fig. 2(b) and supplementary Fig. S3. The peak of $\triangle M({\rm{T}})$ appears exactly at *T*_*w*_, which is the signature of memory effect. Moreover, the peak at *T*_*w*_ becomes sharper and more prominent when the waiting time increases from 1 h to 4 h (Fig. 2(b)), confirming the presence of spin-glass behavior in 18 nm BFO particles. While as shown in the inset of Fig. 2(b) and in our previous work^10^, no memory effect and spin-glass behavior is observed in 45 nm and 83 nm BFO particles. Compared with these larger BFO particles, 18 nm particles possess larger surface-to-volume ratio (supplementary Fig. S4) and thus more uncompensated surface spins due to the broken exchange bonds, which can increase the surface anisotropy of particles^24–26^. At high temperature, thermal energy can override such anisotropy. While as the decrease of temperature, the development of magnetic correlation among surface spins give rise to exchange coupled ferromagnetic clusters whose size increases gradually^26^. That is to say, as shown by the schematic diagram in Fig. 2(c), the FM surface spins of the 18 nm BFO particles can interact with adjacent spins and with the AFM core by interfacial coupling. When the temperature below 35 K (*T*_*B*_), the collective freezing of the surface ferromagnetic clusters occurs, resulting the presence of surface spin-glass state. The rapid increase of remanent magnetization (*M*_*r*_) and coercivity (*H*_*c*_) below *T*_*B*_ (Fig. 3(b)) confirms such variation. Consequently, rapid increased −*H*_*E*_ is observed in 18 nm BFO particles with decreasing temperature from *T*_*B*_, a typical behavior in AFM core/spin-glass shell systems^27,28^. While as the particle size increases, both the number and size of the surface ferromagnetic clusters decrease due to the decreased surface to volume ratio, leading to the weakening of surface anisotropy and the disappearing of the spin-glass behavior (the inset of Fig. 2(b)).

Another interesting phenomenon of 18 nm BFO particles is the nonmonotonic variation of −*H*_*E*_ above *T*_*B*_ (Fig. 1(b,d)), which is different from that obtained in general antiferromagnetic nanostructures, where the value of *H*_*E*_ usually becomes zero above *T*_*B*_ or exhibits monotonic decrease with increasing temperature^15,27,29^. Comparative experiments on BFO particles with various sizes are performed (Fig. 3) to analyze the origin of such nonmonotonic variation. First, as shown in Fig. 3(a), monotonically increased −*H*_*E*_ is observed with decreasing temperature for 45 and 83 nm particles, confirming the peculiar exchange bias behavior of the 18 nm BFO particles. Figure 3(b–d) show the temperature dependent *M*_*r*_ and *H*_*c*_ of 18, 45, and 83 nm BFO particles. It is seen that *M*_*r*_ and *H*_*c*_ decrease obviously for all the particles with temperature decreasing from 300 K to *T*_*B*_. Generally, thermal fluctuation energy decreases with deceasing temperature, which can increase the binding effect of AFM core to FM surface and thus decrease *M*_*r*_ and *H*_*c*_ while increase −*H*_*E*_. While as aforementioned, compared with 45 and 83 nm particles, the interaction among surface spins of 18 nm particles is enhanced, causing that the surface anisotropy increases but the interfacial coupling between the AFM core and FM shell decreases. Moreover, such influence from the interaction of surface spins can increase with decreasing thermal fluctuation, being responsible to the abnormal decrease of −*H*_*E*_ in 18 nm BFO particles as temperature decreases from *T*_*i*_ to *T*_*B*_. Based on this scenario, first, the variation of −*H*_*E*_ should be modulated by cooling magnetic field since the ordered arrangement of surface FM spins can suppress their interaction. As shown in Fig. 1(d), the inflection temperature *T*_*i*_ of −*H*_*E*_, i.e. the starting temperature of −*H*_*E*_ decrease, is decreased from 220 K to 120 K under 65 kOe cooling field, confirming the effect of the interaction of surface spins on exchange bias behavior. Moreover, such influence decreases with increasing particle size. As shown in Fig. 3(a), the increase of −*H*_*E*_ with decreasing temperature is only decelerated obviously below 120 K for 45 nm particles, and such deceleration behavior becomes negligible for 83 nm particles. Second, compared with larger BFO particles, smaller −*H*_*E*_ is observed in 18 nm BFO particles because of its enhanced interaction among surface spins. Finally, for the temperature below *T*_*B*_, the presence of surface spin-glass state of 18 nm BFO particles and the frozen surface spins of the larger BFO particles are responsible respectively for the rapid increase of their *M*_*r*_, *H*_*c*_ and −*H*_*E*_ with decreasing temperature (Fig. 1(b), (d) and Fig. 3). All in all, the surface anisotropy shows great effect on the exchange bias behavior of BFO nanoparticles, especially for the small particles that with enhanced interaction among surface spins. The interplay between the surface ansitropy and the interfacial exchange coupling results in the interesting nonmonotonic variation of −*H*_*E*_ and the surface spin-glass behavior in 18 nm BFO particles.

**Figure 4:**
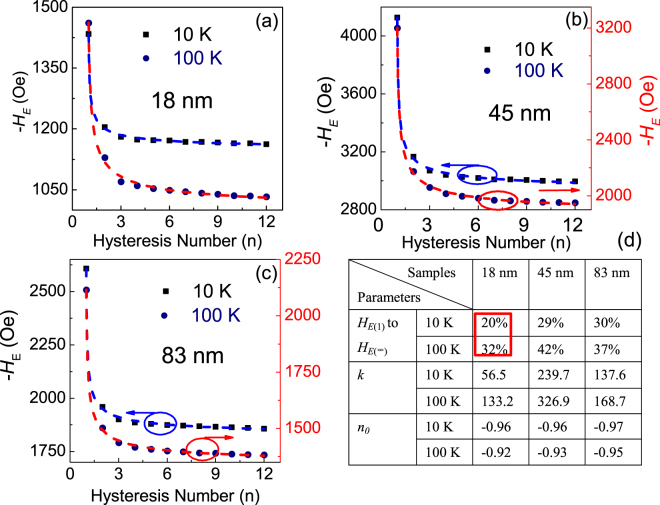
Hysteresis cycles dependent -*H*_*E*_ of BFO particles with size of about (**a**) 18 nm, (**b**) 45 nm and (**c**) 83 nm. (**d**) Fitting parameters by the power-law function.

Figure 4(a–c) shows the dependence of −*H*_*E*_ on the number of repeating cycles (n) at 10 K and 100 K for the BFO particles with size 18, 45 and 83 nm, respectively. It is seen that obvious training effect can be observed in BFO nanoparticles with various sizes, indicating there are still some unstable frozen AFM spins at the interface between AFM core and FM surface, which can be rearranged and eventually approach to equilibrium state. To compare such relaxation behavior of different particles, the training results are fitted by the power-law function^30^, *H*_*E*(*n*)_ = *H*_*E*(∞)_ + *k*(*n* + *n*_0_)^−1/2^, where ${H}_{E(\infty )}$, $k$, ${n}_{0}$ are the fitting parameters. As shown in Fig. 4 by the dashed lines, the experimental results can be fitted well, indicating the energy dissipation of AFM plays a crucial role in the training effect^29^. Meanwhile, as shown in Fig. 4(d), compared with larger particles, the decay from ${H}_{E(1)}$ to ${H}_{E(\infty )}$ of 18 nm BFO particles is improved, implying that the reduced interfacial frozen spins by the interaction of surface spins are the metastable parts. Taken together, in small antiferromagnetic nanoparticles, the correlation among surface spins can give rise to exchange coupled ferromagnetic clusters and enhanced surface anisotropy, which could induce surface spin-glass state and interesting exchange bias behavior and also improve the exchange bias training effect.

## Conclusions

In summary, we have investigated the temperature and size dependent exchange bias behavior of BFO nanoparticles with natural core-shell structure. The 18 nm BFO particles exhibit surface spin-glass state, interesting exchange bias behavior and improved training effect. These results are considered to relate to the enhanced surface anisotropy in small BFO nanoparticles because of their strengthened interaction among surface spins. The present work highlights the role of the surface spin state of antiferromagnetic nanostructures on the modulation of magnetic exchange bias behavior.

## Methods

### Sample preparation

BFO nanoparticles with size from 18 to 83 nm were synthesized by a sol-gel method. The xerogel powders were prepared using the same method as described in our former paper^10^, which were calcined at temperatures 425, 475 and 525 °C for 2 h to obtain BFO nanoparticles with size of about 18 ± 5, 45 ± 7 and 83 ± 20 nm, respectively.

### Magnetic measurements

The magnetic properties of the nanoparticles were measured using a commercial magnetic property measurement system (SQUID-VSM, Quantum Design). For the field-cooling (FC) loops and the training effect measurements, the samples were cooled from 380 K to the required temperature under 65 kOe magnetic field, and the data were recorded thereafter under 50 kOe. It should be noted that such FC process is a little different from the typical process in conventional exchange bias measurement that is usually established by cooling the samples through *T*_*N*_. Here, the BFO nanoparticle is cooled from the maximum temperature 380 K of the instrument, which is much smaller than *T*_*N*_ of BFO. Such decreased onset temperature may decrease the value of exchange bias field but does not affect its variation with temperature. Meanwhile, in order to ensure that there is no trapped flux, samples and superconducting magnet were demagnetized slowly with oscillating field before each measurement.

## Electronic supplementary material

Supplementary Information
