# Supplementary material for: The exchange bias behavior of BiFeO3 nanoparticles with natural core-shell structure
Source: Sci Rep. 2018 Feb 2;8:2311. doi: 10.1038/s41598-018-19676-5 (PMC5797215; doi:10.1038/s41598-018-19676-5)
Supplement: Supplementary file 1 — Supplementary Information [file 41598_2018_19676_MOESM1_ESM.pdf]

Supplementary Information

The exchange bias behavior of $BiFeO_3$ nanoparticles with natural core-shell structure

Fengzhen Huang[1,2], Xingyu Xu[1], Xiaomei Lu[1,2*], Min Zhou[1], Hai Sang[1,2] and Jinsong Zhu[1,2]

[1]National Laboratory of Solid State Microstructures and Physics School, Nanjing University, Nanjing 210093, P. R. China, [2]Collaborative Innovation Center of Advanced Microstructures, Nanjing 210093, P. R. China. Correspondence and requests for materials should be addressed to X.M.L (xiaomeil@nju.edu.cn).

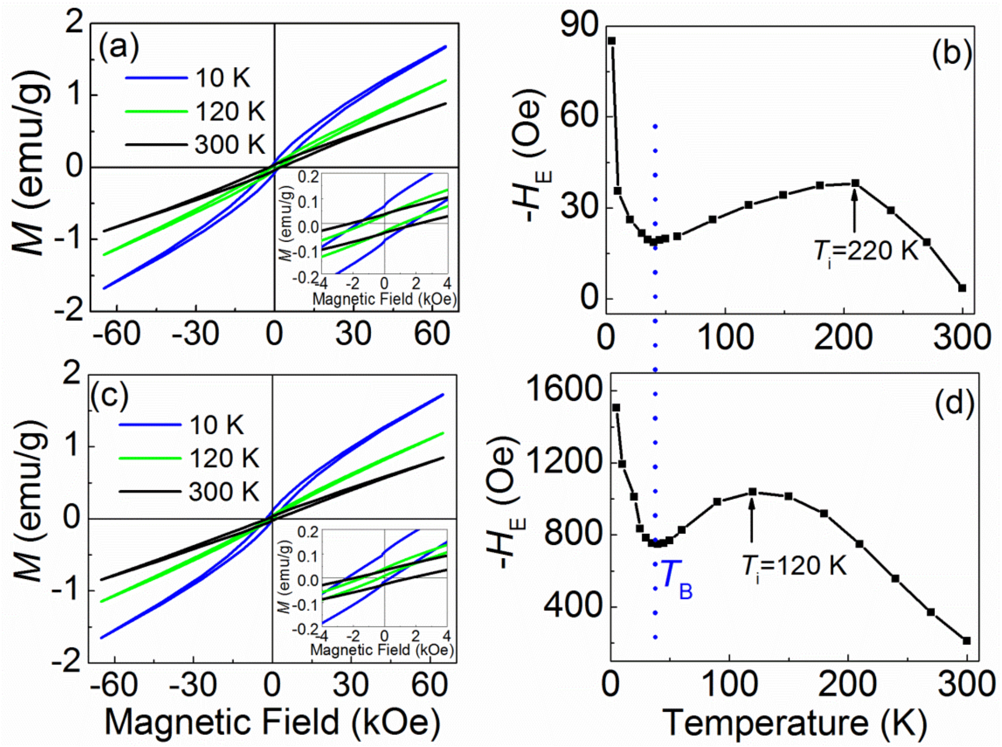

Figure S1. Magnetic measured results of 18 nm $BiFeO_3$ particles with maximum applied magnetic field of 65 kOe. Magnetic hysteresis (*M*-*H*) loops measured at various temperatures following (a) zero-field cooling (ZFC) and (c) 65 kOe cooling field. Temperature dependent exchange bias field ($-H_E$) under (b) ZFC and (d) 65 kOe cooling field. Insets in (a) and (c) show the local amplification of the corresponding *M*-*H* loops.

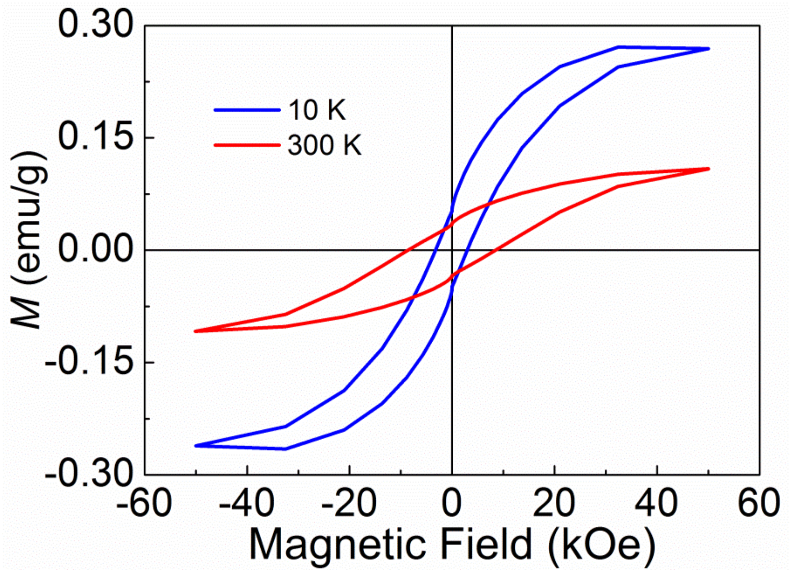

Figure S2. Ferromagnetic parts in the magnetic hysteresis curves of 18 nm $BiFeO_3$ particles at 10 and 300 K.

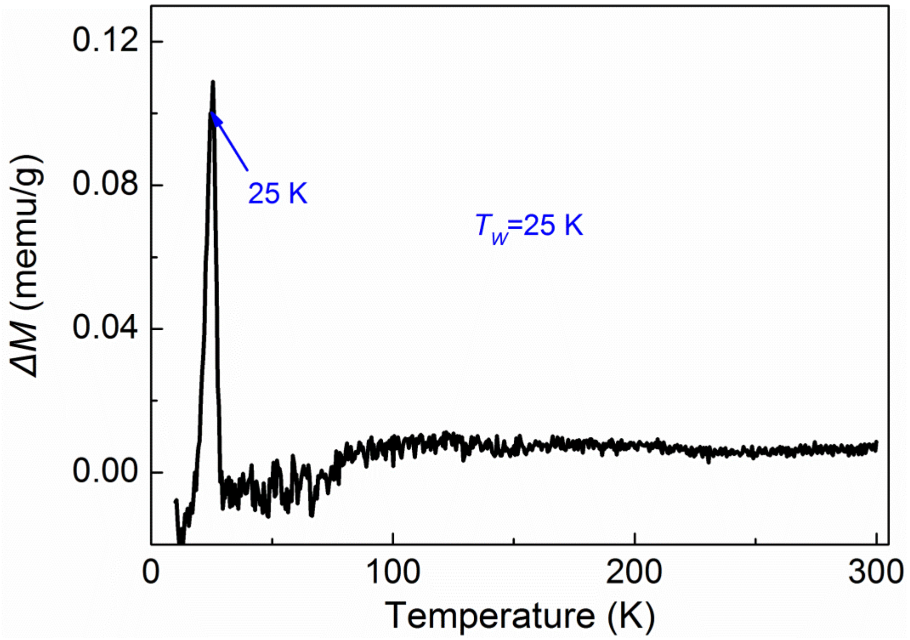

Figure S3. Magnetization difference (ΔM) between the reference and the stop-and-wait ZFC curves of 18 nm $BiFeO_3$ particles, where $T_w = 25$ K, and the waiting time is 4 h.

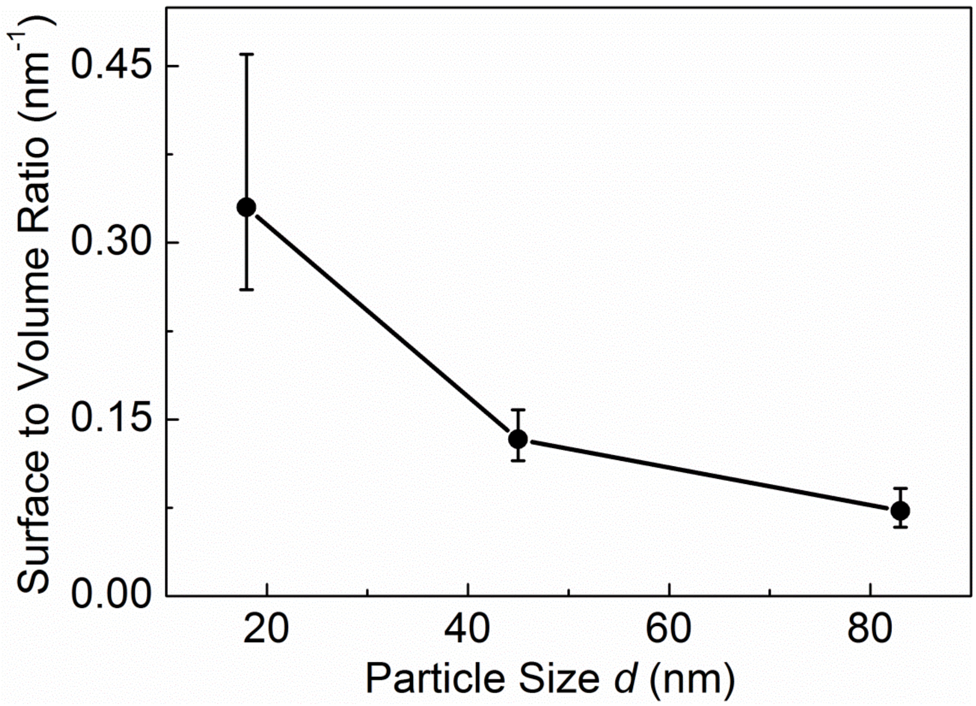

Figure S4. Surface-to-volume ratio of $BiFeO_3$ nanoparticles with various sizes (*d* is the diameter of the particle), which is calculated based on spheroidal particle model.
